# The Resistance Paradox in COVID-19 Ventilator-Associated Pneumonia: A Retrospective Study on Rapid Molecular Stewardship

**DOI:** 10.3390/antibiotics15030236

**Published:** 2026-02-24

**Authors:** Andrei Mihai Bălan, Tudor-Mihai Magdaș, Andrada Elena Urda-Cîmpean, Constantin Bodolea, Andrada Nemeș, Lucreția Avram, Dana Crișan, Sebastian Trancă

**Affiliations:** 1 2nd Department of Anesthesia and Intensive Care, “Iuliu Hatieganu” University of Medicine and Pharmacy, 8 Victor Babeş Street, 400012 Cluj-Napoca, Romania; balan.andrei@umfcluj.ro (A.M.B.); tranca.sebastian@umfcluj.ro (S.T.); 2 Department of Anesthesia and Intensive Care, Municipal Clinical Hospital, 400139 Cluj-Napoca, Romania; 3 Department of Medical Informatics and Biostatistics, “Iuliu Hatieganu” University of Medicine and Pharmacy, 8 Victor Babeş Street, 400012 Cluj-Napoca, Romania; aurda@umfcluj.ro; 4 Geriatrics—Gerontology, Department 5—Medical Specialties, Faculty of Medicine, Iuliu Haţieganu University of Medicine and Pharmacy, 400012 Cluj-Napoca, Romania; 5 Department of Internal Medicine, 5th Medical Clinic, Clinical Municipal Hospital, Faculty of Medicine, “Iuliu Hatieganu” University of Medicine and Pharmacy, 400012 Cluj-Napoca, Romania; 6 Emergency Department, The Emergency County Hospital Cluj, 400347 Cluj-Napoca, Romania

**Keywords:** critical care, antibiotic stewardship, Point-of-Care Polymerase Chain Reaction, POC-PCR, multidrug resistance, Ventilator-associated pneumonia, COVID-19 infection

## Abstract

**Background/Objectives:** The COVID-19 pandemic complicated the diagnosis of Ventilator-Associated Pneumonia (VAP), leading to empiric antibiotic overuse due to the difficulty in distinguishing viral progression from bacterial superinfection. However, it remains unclear whether COVID-19-associated VAP displays a distinct antimicrobial resistance profile compared to classical VAP. **Methods:** This monocentric, retrospective cohort study primarily investigated differences in clinical phenotypes and antibiotic resistance profiles between patients with VAP-COVID (n = 26) and non-COVID-VAP (n = 26). Logistic regression was used to identify factors associated with the COVID-19 phenotype and predictors of antimicrobial resistance. As a secondary objective, we evaluated the diagnostic efficacy of a multiplex Point-of-Care PCR (POC-PCR) system (n = 22) compared to standard culture (n = 26) regarding turnaround time and resistance detection. **Results:** Patients with VAP-COVID exhibited significantly higher resistance rates to carbapenems (76.9% vs. 50%, *p* = 0.04) and fluoroquinolones (88.5% vs. 61.5%, *p* = 0.02) despite fewer traditional risk factors at admission. The clinical profile of the VAP-COVID group was distinguished by a significantly lower incidence of parapneumonic pleural effusion (19.2% vs. 84.6%, *p* < 0.001) and a higher median Neutrophil-to-Lymphocyte Ratio (41.36 vs. 9.63, *p* < 0.001). Regarding diagnostic speed, POC-PCR significantly reduced the time to result validation compared to standard culture (~1 h vs. ~62.5 h, *p* < 0.001). **Conclusions:** VAP in COVID-19 patients presents a distinct microbiological profile characterized by higher antimicrobial resistance. In this context, the integration of rapid molecular diagnostics may support earlier microbiological guidance compared to standard methods.

## 1. Introduction

The COVID-19 pandemic rapidly emerged as a healthcare emergency. Although the acute phase of the pandemic has subsided, we must acknowledge that the impact of the crisis extended beyond immediate mortality. While a total of 5.94 million deaths were reported between January 2020 and December 2021, excess mortality estimates suggest a toll as high as 18.2 million [1].

Ventilator-associated pneumonia (VAP) is defined as pneumonia that occurs in patients who require mechanical ventilation for more than 48 h. It is one of the most common infectious complications in critical care and is associated with increased morbidity and mortality [2,3,4,5,6,7]. The incidence of VAP increased significantly during the pandemic, with reported rates reaching up to 50% [8,9,10,11,12,13,14,15,16,17,18,19]. This increase was caused by various factors: intrinsic pathophysiological issues, such as extensive lung damage and immune dysregulation, and operational challenges, like staffing shortages and inadequate personal protective equipment (PPE), which led to cross-contamination [14,20,21,22,23,24,25,26,27,28]. The clinical presentation of COVID-19 often included severe hypoxemia, leukocytosis, and extensive bilateral infiltrates, which greatly overlap with bacterial superinfection. Traditional diagnostic criteria have proven unreliable in COVID-19 cases, severely impairing accurate diagnosis and stewardship efforts [29,30].

This diagnostic ambiguity led to the overuse of broad-spectrum empiric antibiotics despite the absence of a confirmed bacterial cause [31,32]. This approach conflicts with the core principles of effective stewardship and has altered the resistance patterns of multiple pathogens [33,34,35]. Although the acute viral surge has subsided, the diagnostic challenges identified remain highly relevant to current practice, where distinguishing viral inflammation from bacterial sepsis continues to be a daily ICU challenge.

This study examined the distinct clinical and microbiological phenotypes of COVID-19 VAP compared to VAP non-COVID-19. We examined the microbiological resistance patterns, according to international guidelines for the responsible use of antimicrobials, to specifically characterize the surge in antimicrobial resistance under the pandemic operational pressures [32,36,37].

Additionally, we conducted a secondary sub-analysis to compare the turnaround time and effectiveness of Point-of-Care PCR (POC-PCR) with standard microbiological culture to assess its role as a key stewardship tool.

## 2. Results

The findings presented should be interpreted as exploratory and hypothesis-generating. Given the limited sample size and the presence of small cell counts in several comparisons, the results are susceptible to random variation and statistical instability. Accordingly, the primary objective of this analysis is not to establish definitive causal inferences but rather to describe and characterize the clinical and microbiological profiles of patients in the COVID-19 and historical control cohorts, and to identify potential signals that warrant further investigation in larger, adequately powered studies.

### 2.1. Patient Characteristics

A total of 52 patients were included in the study and divided into two analysis groups: VAP-COVID (n = 26) and VAP non-COVID (n = 26). Baseline demographics (age, sex) were similar, but the admission diagnoses differed significantly between groups. The primary admission diagnosis in the VAP-COVID group was acute respiratory failure (96.15%), whereas the VAP non-COVID group mainly presented with septic shock (61.53%) and altered neurological status (53.84%).

Baseline comorbidities differed significantly (Table 1). In the VAP-COVID group, there was a higher prevalence of Diabetes Mellitus (50% vs. 23.1%, *p* = 0.044). Conversely, the VAP non-COVID group showed higher rates of ischemic heart disease (*p* = 0.020), vascular disease (*p* = 0.016), and gastroesophageal reflux disease (GERD) (*p* = 0.010).

### 2.2. Risk Factors and Clinical Presentation

The VAP non-COVID group showed a higher prevalence of traditional risk factors for multidrug-resistant (MDR) infections, including septic shock at admission (*p* < 0.0001), recent hospitalization (*p* = 0.008), and recent antibiotic exposure (*p* = 0.008) (Table 2). The specific location of previous hospitalizations could not be verified due to the unavailability of comprehensive medical records, which may have influenced the selection of initial empirical antimicrobial therapy for these patients.

The VAP-COVID group had higher rates of iatrogenic risk factors for VAP, such as increased exposure to systemic corticosteroids (96.2% vs. 26.9% in the non-COVID group, *p* < 0.001) and proton pump inhibitors (84.6% vs. 19.2%, *p* < 0.001) (Table 2).

Respiratory support before intubation in the ICU also differed (Table 2). At admission, 69.2% of VAP-COVID patients required noninvasive ventilation (NIV/CPAP), whereas 53.8% of VAP non-COVID patients were already invasively ventilated (*p* < 0.001). Immediately prior to intubation, 88.5% of the VAP-COVID group were on NIV, compared to only 42.3% of the non-COVID group (*p* < 0.001).

We observed notable differences in the initial antimicrobial management. The VAP-COVID group was primarily treated with Ceftriaxone (73%) or Doxycycline (34.6%), whereas the VAP non-COVID group received broad-spectrum antibiotic combinations (e.g., Vancomycin or Linezolid combined with Gram-negative agents) (23% vs. 3.84%, *p* = 0.04).

### 2.3. VAP Diagnostic Findings

Radiological patterns varied between groups (Table 3). In the non-COVID group, 100% of VAP cases were identified by new lung consolidations, while VAP-COVID patients exhibited more ambiguous, progressive infiltrates (42.3%) or mixed patterns. Additionally, parapneumonic pleural effusion was observed in 84.6% of non-COVID patients but was absent in the VAP-COVID group (19.2%; *p* < 0.001).

Septic shock was significantly more common in the VAP non-COVID group (92.3% vs. 61.5%, *p* = 0.008) at the time of VAP diagnosis. Clinical criteria, such as leukocytosis, purulent secretions, or worsening oxygenation, were present in similar proportions in both groups (Table 3).

The average laboratory turnaround time for bacteriological validation was notably longer for the VAP-COVID group compared to the VAP non-COVID group, but the VAP-COVID group spent statistically detectable fewer days in different stages and developed VAP quicker than the VAP non-COVID group (Table 3).

### 2.4. Microbiology and Antimicrobial Resistance

The most common causes of VAP were *Acinetobacter baumannii*, *Klebsiella pneumoniae*, and *Pseudomonas aeruginosa* in both study groups. *Escherichia coli* and *Staphylococcus aureus* (MRSA and MSSA) were detected more often in the VAP non-COVID group. Fungal co-infections (*Candida* spp.) occurred more frequently in the VAP-COVID group (26.9% vs. 11.5%). Higher resistance rates were observed in the VAP-COVID group, with increased resistance to carbapenems (76.9% vs. 50%, *p* = 0.04) and fluoroquinolones (88.5% vs. 61.53%, *p* = 0.02). Unadjusted odds ratio (OR) for Carbapenem Resistance rate in VAP-COVID vs. non-COVID was 3.33 (95% CI [1.01; 11]). Unadjusted OR for Fluoroquinolone Resistance rate in VAP-COVID vs non-COVID was 4.79 (95% CI [1.14; 20.2]). Both results suggest higher odds in the COVID cohort, but confounding is possible due to baseline severity differences. In early-onset VAP (≤5 days), carbapenem resistance was even higher in the VAP-COVID group (80% vs. 36.36%, *p* = 0.043).

Empiric antibiotic therapy initiated before microbiological results was inadequate in a high proportion of patients from both groups (88.46% VAP-COVID vs. 73% VAP non-COVID, *p* = 0.15) (Table 4).

We analyzed multiple logistic regression models to evaluate the potential drivers of carbapenem resistance. The first model, which incorporated the study group, SAPS II scores, and the Charlson Comorbidity Index, was statistically significant (χ^2^(3) = 10.793, *p* < 0.05) and explained 20.2% (Nagelkerke R2) of the variance. Despite the overall model significance, no individual factor reached statistical threshold in this analysis. A subsequent model, which substituted the comorbidity index with septic shock at admission, also achieved significance (χ^2^(3) = 10.793, *p* < 0.05). This version explained 25.6% of the variance and correctly classified 81.8% of cases, revealing that lower SAPS II values at admission were associated with a marginally higher likelihood of carbapenem resistance (OR: 0.95, 95% CI [0.91; 0.99]).

In contrast, the regression models for fluoroquinolone resistance did not achieve statistical significance. A model including the study group, SAPS II, and the Charlson Comorbidity Index reached a significant chi-square (χ^2^(3) = 10.793), explaining only 16.5% of the variance with no significant predictors. Similarly, a model adjusting for study group, SAPS II, and septic shock was found to be non-significant (χ^2^(3) = 7.621, *p* > 0.05), accounting for 20.2% of the variance in fluoroquinolone resistance without reaching statistical thresholds for individual factors.

### 2.5. Clinical Outcomes and Prognostic Scoring

**Figure 1:**
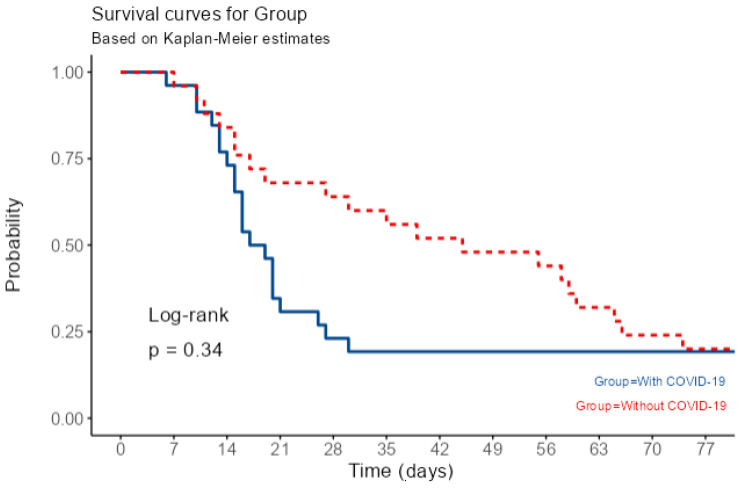
Kaplan–Meier survival curves for VAP-COVID (blue) and VAP non-COVID (red) cohorts. Median survival times differed numerically (18 days vs. 45 days), but the survival distributions did not differ significantly (log-rank *p* = 0.34).

Overall ICU mortality was high and similar between the two groups (80.8% VAP-COVID versus 88.5% VAP Non-COVID; *p* = 0.442, Chi-square test). Kaplan–Meier analysis revealed different mortality patterns. The VAP-COVID group had a shorter median survival of only 18 days (95% CI: 15–27 days), compared to 45 days (95% CI: 27–66 days) in the VAP Non-COVID group. Survival rates were similar only during the first week (~96%). After this initial period, the VAP-COVID curve declined sharply, and by Day 21, survival dropped to 30.8%, versus 68.0% in the Non-COVID group. Overall mortality was similar (>80% in both groups) due to late deaths in the Non-COVID group. Therefore, overall survival did not differ significantly between groups (Log-rank *p* = 0.34) (Figure 1).

The progression of this disease in the VAP-COVID cohort was linked to the resistance profile analysis. In the VAP-COVID group, patients with Extensively Drug-Resistant (XDR) organisms experienced the highest early mortality rates, with 40% of deaths happening between days 14 and 28. In opposition, in the VAP Non-COVID group, XDR-related deaths mainly occurred later, with most (70.6%) happening after Day 28. The overall mortality rate in the sample was a bit smaller in the VAP-COVID group than the VAP-COVID group, but with no detectable significance (OR = 0.55 95% CI [0.11; 2.58]).

VAP non-COVID patients had longer survival times with no detectable significance, but with significantly more total hospital days (average 43.3 vs. 17.4 days, *p* = 0.004) and longer mechanical ventilation durations (average 24.2 vs. 13.3 days, *p* = 0.001).

**Figure 2:**
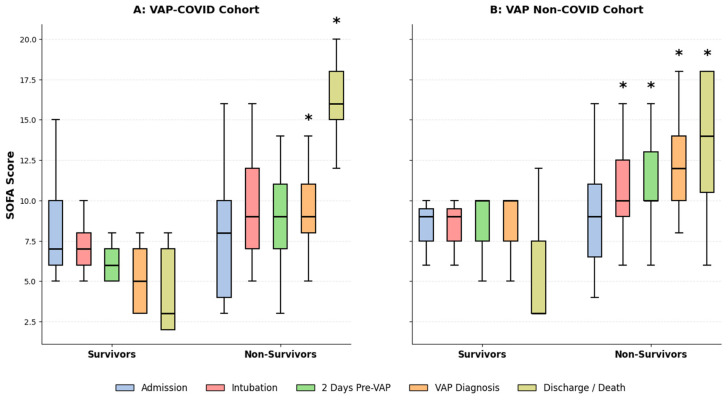
Dynamic evolution of SOFA scores stratified by survival status in (**A**) VAP-COVID and (**B**) VAP non-COVID patients. The plots show the median SOFA scores with interquartile ranges at five critical clinical stages. (**A**) In the VAP-COVID cohort, non-survivors experienced a sharp, significant increase in organ failure scores, especially from VAP diagnosis to death. (**B**) Conversely, the VAP non-COVID cohort demonstrated a gradual rise in scores across all time points in non-survivors. (*) Indicates a statistically significant difference (*p* < 0.05) compared to the previous clinical stage.

The evolving pattern of organ failure scores was associated with mortality (Figure 2; Table 5). Patients in the VAP non-COVID group had higher Charlson Comorbidity Index (CCI; 8.16 vs. 4.77; *p* < 0.001) and higher baseline SOFA scores two days before VAP diagnosis (10.85 vs. 8.47; *p* = 0.008). In contrast, VAP-COVID patients had lower assessment scores until superinfection occurred. From the point of VAP diagnosis, non-survivors in the VAP-COVID group exhibited a sharp, significant linear increase in SOFA scores leading up to death (*p* < 0.05).

A logistic regression was performed to o ascertain the effects of group, SAPS II and Charlson Comorbidity Index at admission on the likelihood that participants would die. The logistic regression model was not statistically significant, χ^2^(2) = 2.492, *p* > 0.05.

### 2.6. Hematological Profile of Deceased Patients

In a sub-analysis restricted just to deceased patients (VAP-COVID n = 21; VAP non-COVID n = 23), notable differences in hematological profiles were observed, while age and platelet counts remained similar (Table 6). At ICU admission, the VAP-COVID group already showed significant lymphopenia (median 0.44 vs. 0.68 K/µL, *p* = 0.019). This difference became more pronounced at the time of VAP diagnosis, with the VAP-COVID group exhibiting a different inflammatory response, characterized by significantly higher neutrophils (median 15.63 vs. 10.04 K/µL, *p* < 0.001) and persistently lower lymphocytes (median 0.46 vs. 0.77 K/µL, *p* = 0.004). Consequently, the Neutrophil-to-Lymphocyte Ratio (NLR) was significantly higher in the VAP-COVID group compared to the non-COVID group (median 41.36 vs. 9.63, *p* < 0.001). NLR was also significantly elevated at Day 28 (*p* < 0.001). Conversely, the VAP non-COVID group consistently had higher counts of monocytes, eosinophils, and basophils, along with a significantly higher RDW-SD at all three measured time points (*p* < 0.001).

### 2.7. Rapid Molecular Diagnostic Sub-Analysis (POC-PCR)

To assess potential solutions to diagnostic delays, we compared a subgroup of VAP patients diagnosed with Point-of-Care PCR (POC-PCR; n = 22) with those diagnosed with standard culture (n = 26). Baseline demographic characteristics and severity scores (SOFA, APACHE II) were similar between these subgroups.

The analysis demonstrated that POC-PCR significantly reduced the mean time to result validation to 61.31 ± 9.87 min, compared with 3752.72 ± 1675.45 min (~62.5 h) for standard culture (*p* < 0.001). POC-PCR detected resistance markers not identified by phenotypic testing, yielding a significantly higher detection rate of Colistin resistance than standard methods (36.4% vs. 3.8%; *p* = 0.003).

## 3. Discussion

### 3.1. The Diagnostic Ambiguity

Effective antimicrobial management relies on a physician’s ability to distinguish bacterial superinfection from viral progression. In the context of COVID-19, however, traditional clinical and radiological signs are unclear, leading to antibiotic overuse despite low confirmed bacterial causes [10,30,38,39].

The progressive consolidations observed radiologically in SARS-CoV-2 infection, present in 42.3% of the cohort, reflect specific pathophysiological mechanisms such as immunothrombosis and pulmonary infarction, rather than bacterial consolidation [31]. Notably, another classic sign of bacterial pneumonia, the parapneumonic pleural effusion, was less common in the VAP-COVID group than in the non-COVID controls (19.2% vs. 84.6%), consistent with the literature and indicating that its presence predicts the absence of COVID-19 pneumonia [40,41]. Although the type of sampling can affect the power of the microbiological part of the diagnostic process, in this study, only classic sampling from the endo-tracheal tube was used due to the lack of staff competencies and lack of specific equipment (bronchoscopes). This could have led to contamination and could be a reason for over-diagnosis.

To further complicate diagnosis, biological markers such as C-Reactive Protein and Procalcitonin were not reliable in distinguishing overlapping bacterial infections in COVID-19 [42,43,44]. This altered inflammatory response results from both the viral infection itself and from the immunomodulatory treatments used, like dexamethasone and tocilizumab [4,5,16,42,43,44,45,46]. Therefore, to overcome the limitations of subjective clinical judgment and improve prescription accuracy, diagnostic-guided algorithms are essential, as emphasized by the European Society of Clinical Microbiology and Infectious Diseases (ESCMID) [36].

### 3.2. The Impact of Empiric Antibiotic Overuse

Local microbiological patterns remain a key factor in VAP etiology and cause significant variation across different centers [4,5,46]. In our study group, consistent with the broader literature, the main pathogens of COVID-19 VAP were Gram-negative bacteria—*Enterobacteriaceae*, *Acinetobacter baumannii*, and *Pseudomonas aeruginosa* [16,19,47,48,49]. In our study, the VAP-COVID group had notably fewer traditional admission risk factors but experienced significantly higher rates of Carbapenem resistance (76.9% vs. 50%, *p* = 0.04) and fluoroquinolone resistance (88.5% vs. 61.5%, *p* = 0.02) compared to historical controls. These resistance rates likely reflect a multi-factorial mechanism, driven by iatrogenic pressures such as almost universal use of systemic corticosteroids (96.2%) and PPIs (86.4%), with consequent immunosuppression and prolonged non-invasive ventilation time (88.5%), and the pandemic associated operational strain. Early intervention, meticulous monitoring and personalized care are paramount for enhancing survival in SARS-CoV-2 pneumonia, but due to staffing shortages, high ICU workloads and the lack of protective equipment (PPE), the prevention of secondary hospital-acquired infections was likely hindered [50].

Prudent antibiotic use is essential to prevent MDR development [51]. Guidelines advise against antibiotic use in patients with negative cultures, but due to the diagnostic challenges associated with COVID-19 infection and the fear of missing a bacterial superinfection, widespread empirical antibiotic use was observed from the start of treatment [14,52,53]. A notable example is the heavy reliance on azithromycin during early pandemic waves, a common practice later disproven but still contributing to the selective pressure for MDR [52,54].

In cohorts where patients with an unconfirmed diagnosis of pneumonia received broad-spectrum antibiotics, a resistant phenotype emerged, with carbapenem resistance rates reaching as high as 100% for *Acinetobacter* isolates, and a significant rise in high-risk clones carrying *blaOXA-23* and *blaNDM* genes [55,56,57,58].

Iatrogenic factors specific to pandemic protocols further increased this risk. The widespread use of systemic corticosteroids (96.2%) and proton pump inhibitors (PPIs) (84.6%) in our VAP-COVID group may have contributed to secondary infections. Corticosteroids are known to promote VAP development by affecting the immune response [59,60], and high PPI use causes hypochlorhydria and colonization of the aerodigestive tract [61,62]. Prolonged Non-Invasive Ventilation (NIV), used frequently in our cohort (88.5% of VAP-COVID patients), is a risk factor for pulmonary microaspiration [31]. Additionally, longer laboratory turnaround times contributed to the emergence of resistant phenotypes by extending the period of inadequate empiric therapy, which occurred in 88.5% of our VAP-COVID cases [29,63].

This resistance paradox represents a lasting “ecological scar,” not just a temporary anomaly from the past crisis [55]. It is reported that resistance phenotypes have not reverted to pre-pandemic levels but have stabilized at dangerous new highs [64,65,66]. Antunes et al. reported that resistance rates in *Klebsiella pneumoniae* doubled during the surge (from 26% to 51%) and remained high at 42% post-pandemic. Additionally, carbapenem resistance in Acinetobacter baumannii surged to 92% and settled at 85% after the surge, far above pre-pandemic levels [64]. In China, resistance in *Carbapenem-Resistant K. pneumoniae* (CRKP) to monobactams peaked at 96.9% in 2024 [65]. Turkish data further reinforce this trend, showing significant post-pandemic increases in resistance of *Acinetobacter* and *Klebsiella* to gentamicin and colistin [66].

This landscape has become the new norm for MDR microorganisms, as empirical coverage shifts with the expansion of hypervirulent lineages [67]. Given the high resistance levels and diagnostic uncertainties, implementing vigilance tools is essential. Validated risk stratification methods, such as the Clinical Pulmonary Infection Score (CPIS), along with high-sensitivity rapid molecular tests, are necessary to enable targeted therapy [64].

### 3.3. Rapid Molecular Diagnostic Sub-Analysis

Our data may indicate that POC-PCR offers quick microbiological insights that can support early treatment decisions in the ICU, as it considerably shortened the diagnostic turnaround time from 62 h to just 1 h [68,69,70]. This practice helps meet the World Health Organization’s standards for prompt diagnosis to guide antimicrobial therapy and aligns with the European Commission’s goal to implement rapid diagnostics that aid clinical decision-making and reduce unnecessary antimicrobial use [37,71].

Molecular tools have higher diagnostic sensitivity, allowing for detection of pathogens and resistance markers that standard cultures may miss. This enables the clinical team to initiate targeted antibiotics within the first hour of suspicion [29]. Therefore, these tools provide a promising way to safely shorten antibiotic courses, reduce overuse, and facilitate early de-escalation [72]. Recent data confirms that implementing such diagnostic bundles significantly decreases broad-spectrum antibiotic use while maintaining similar clinical outcomes, establishing POC-PCR as a vital tool in ICU care [73]. These findings could impact antimicrobial stewardship by enabling earlier microbiological guidance.

### 3.4. Limitations

Our study recognizes several limitations. First, it is a retrospective, single-center study with a relatively small sample size, which inherently limits its statistical power, generalizability of the findings and causal inference. The absence of data regarding antibiotic administration regimes makes the relationship between empiric overuse and the microbial resistance largely inferential. We acknowledge that microbial ecology is institution-specific and that resistance rates reported here represent site-specific data rather than a universal overview applicable to other ICUs. Consequently, our results are intended to be exploratory and highlight trends, but we recognize that broader multicenter prospective studies are required to ensure generalizability. Comparing a pandemic cohort to a historical control may also introduce temporal bias in clinical practices over time, but matching criteria and statistical significance support our findings. Furthermore, the use of a historical control group introduces inherent temporal bias, and changes in ICU practices and antimicrobial stewardship strategies may account for some differences observed. Additionally, due to the small sample size, there were not enough patients with concomitant chronic key conditions to support a statistically significant analysis. We also acknowledge the risk of selection bias, as the patients were included only if complete microbiological data were available, potentially overestimating resistance rates, mortality or the proportion of inadequate empiric therapy.

The main limitation of rapid molecular tests is the inability to distinguish between bacterial colonization and active infection organisms [74,75,76]. In our study, we lacked a specific assessment of fungal infection and viral reactivations to delimitate a clear distinction between colonization and invasive disease.

The study may also be affected by selection bias, as patients were included only if complete microbiological data were available. This approach may preferentially include patients with more severe disease or longer ICU stays, potentially inflating resistance rates, mortality, and the proportion of inadequate empiric therapy. Additionally, our study did not measure if employing the rapid molecular tests translated into direct clinical benefits, such as earlier antibiotic de-escalation or improved survival.

Furthermore, our study lacks diagnostic depth regarding fungal and viral co-pathogens. While Candida species were detected, the retrospective design prevented us from definitively distinguishing between airway colonization and invasive fungal disease, as specialized biomarkers or histopathological data were unavailable. Additionally, we did not explore viral reactivations, such as *Herpes Simplex Virus* (HSV) or *Cytomegalovirus* (CMV), which are increasingly recognized as contributors to ICU morbidity and may have confounded our inflammatory markers and outcome measures. The high rates of systemic inflammation observed may therefore reflect a more complex interplay of multiple infectious drivers than currently captured in our microbiological analysis.

### 3.5. Future Perspectives for the Post-Pandemic Era

The persistence of high-level resistance phenotypes, such as sustained carbapenem resistance in *Acinetobacter* spp. and *Klebsiella* spp., suggests that traditional “escalation” strategies may now carry increased risks of early treatment failure [37,65,66,67,68,69,70,71,72,73,74,75,76,77]. As the current microbiological landscape has changed significantly, future research focused on measuring the reduction in empiric antibiotic days could provide the necessary clinical and economic justification. There is a need to determine if the integration of POC-PCR translates clinically to a reduction in antibiotic days and lower rates of resistance emergence.

Also, as emerging post-pandemic data identified a ‘resistance paradox’, universal conclusions require a larger number of patients to ensure representativeness through future multicenter prospective studies and meta-analyses.

## 4. Materials and Methods

### 4.1. Study Design and Rationale

We conducted a retrospective observational study in the ICU of Cluj-Napoca Municipal Clinical Hospital. The study aimed to: (1) characterize the clinical and microbiological profile of VAP in COVID-19 patients compared to historical controls, and (2) compare the turnaround time and resistance detection performance of Point-of-Care PCR versus traditional microbiological culture. The study protocol adhered to the ethical guidelines of the 1975 Declaration of Helsinki and was approved by the Hospital Ethics Committee (Approval No. 7/2020).

### 4.2. Study Population and Cohort Stratification

**Figure 3:**
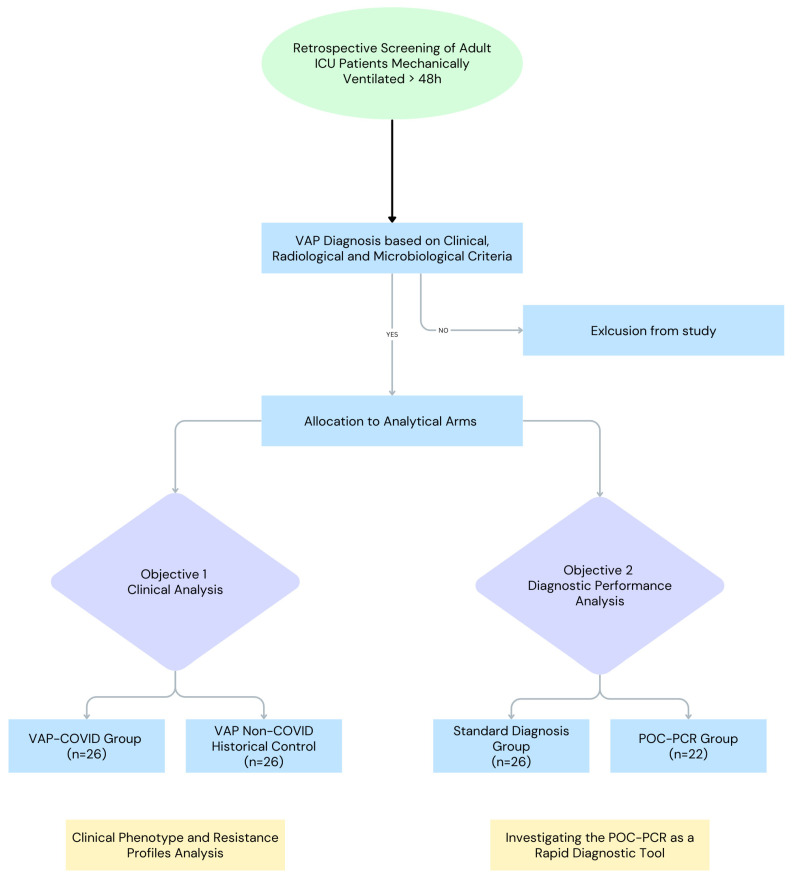
Study design and patient allocation flowchart. Patients were stratified into two analytical arms: (Left) A clinical phenotype analysis comparing pandemic vs. historical cohorts, and (Right) A diagnostic performance sub-analysis comparing standard culture vs. Point-of-Care PCR.

We included adult patients (>18 years) who were mechanically ventilated for at least 48 h and developed Ventilator-Associated Pneumonia (VAP). Patients with early mortality (<48 h after admission) were excluded. Patients were selected consecutively based on the availability of complete microbiological data. After retrospective chart screening, patients were assigned to two distinct analytical groups (Figure 3):

Objective 1: Clinical and Microbiological Phenotype Analysis

To assess the impact of the pandemic on VAP outcomes and resistance, we compared:

- VAP-COVID Group (n = 26): Patients admitted during the pandemic (2020–2021) with severe SARS-CoV-2 pneumonia and confirmed VAP.
- VAP non-COVID historical Control (n = 26): A historical cohort with comparable baseline characteristics admitted in the pre-pandemic period (January 2018–January 2020) with VAP.

Objective 2: Diagnostic Performance Analysis

To evaluate the usefulness of molecular diagnostics outside of pandemic-related operational limitations, we studied a separate group of patients from the pre-pandemic period. We used this study design to avoid the confounding effects of COVID-19 isolation protocols.

- Standard Diagnostics Group (n = 26): Patients diagnosed exclusively via standard culture and antibiogram (disk diffusion/automated systems).
- POC-PCR Group (n = 22): Patients diagnosed using standard culture combined with a multiplex Point-of-Care PCR system.

### 4.3. Definitions and Criteria

VAP diagnosis was established according to international guidelines [4,5]. Only the first episode of VAP for each patient was included in the analysis. A confirmed case required the presence of a new or worsening radiological infiltrate combined with at least two clinical signs of infection: fever > 38.3 °C, purulent tracheal secretions, or leukocytosis (>12,000/µL) or leukopenia (<4000/µL). Microbiological diagnosis was confirmed with a positive quantitative culture (≥10^5^ CFU/mL) from endotracheal aspirates. Endotracheal aspiration was the sampling technique of choice, as fiberoptic bronchoscopy for bronchoalveolar lavage was not available during the study period. Resistance profiles were classified as Multidrug-Resistant (MDR), Extensively Drug-Resistant (XDR), or Pandrug-Resistant (PDR).

### 4.4. Data Collection

For the clinical analysis, we documented demographics, comorbidities (Charlson Index), severity scores (SOFA, APACHE II, SAPS II), and treatment data (corticosteroids, gastric suppression). The SOFA score was monitored dynamically at admission, intubation, 2 days before VAP, at VAP diagnosis, and at discharge to assess ongoing organ failure. For the diagnostic sub-analysis, we specifically recorded the time to result validation (measured in minutes from sampling to the identification of the pathogen) and the concordance of resistance detection between PCR and culture.

### 4.5. Statistical Analysis

All statistical analyses presented in the results section are exploratory, no corrections were made despite low sample size. Continuous variables were reported as mean ± standard deviation (SD) or median with interquartile range (IQR) for non-normally distributed data, while categorical variables are shown as frequencies (n) and percentages (%). Statistical analysis was performed using Student’s *t*-test or the Mann–Whitney U test for continuous variables, and to estimate effect sizes we computed Standardized Mean Differences (Cohen’s *d*) and 95% confidence intervals. For the categorical variable, we used the Chi-square test or Fisher’s exact test, and to estimate effect sizes we computed Standardized Mean Differences (SMD) for binary variables. For survival analysis, we employed Kaplan–Meier curves with Log-rank testing. A *p*-value < 0.05 was considered statistically significant. Statistical analyses were conducted using SPSS v.28.0 (IBM Corp., Armonk, NY, USA), Jamovi v. 2.3 (The jamovi project (2022) https://www.jamovi.org, accessed on 10 February 2026) using R: A Language and environment for statistical computing v 4.1 (R Core Team (2021) https://cran.r-project.org, accessed on 10 February 2026) and Microsoft Excel (Microsoft Office Professional Plus 2021).

## 5. Conclusions

Aligning with emerging global post-pandemic data, we identified a distinct COVID-19 VAP phenotype characterized by iatrogenic risk factors, diagnostic ambiguity, and a “resistance paradox,” where patients exhibit high carbapenem resistance despite fewer traditional risk factors. To address the emergence of this high-resistance landscape, our analysis confirms that implementing Point-of-Care PCR reduces diagnostic turnaround time by over 60 h compared to standard culture. The integration of rapid molecular diagnostics may support earlier microbiological guidance in an era of diagnostic uncertainty. These exploratory findings support further prospective studies to clarify the clinical impact of early microbiological information in critically ill patients with VAP.

## Figures and Tables

**Table 1:** Patient demographics, admission etiology, and baseline comorbidities. Data presented as frequency n (%) or Mean ± Standard Deviation. GERD: Gastroesophageal Reflux Disease. SMD: Standardized Mean Difference.

Variable	VAP-COVID (n = 26)	VAP Non-COVID (n = 26)	*p*-Value	SMD (95% CI)
Age (Mean ± SD)	69.0 ± 11.37	73.42 ± 9.08	0.07	−0.43 [−0.99; 0.14]
Sex—Male (%)	17 (65.4%)	18 (69.2%)	0.76	−0.081
Primary Admission Reason				
Acute Respiratory Failure	25 (96.15%)	13 (50%)	<0.001	1.218
Septic Shock	4 (15.38%)	16 (61.53%)	<0.001	−1.078
Altered Neurologic Status	8 (30.76%)	14 (53.84%)	0.08	−0.480
Comorbidities				
Diabetes Mellitus	13 (50%)	6 (23.1%)	0.044	0.582
Chronic Ischemic Heart Disease	13 (50%)	21 (80.8%)	0.020	−0.684
Vascular Disease	4 (15.4%)	12 (46.2%)	0.016	−0.708
GERD	0 (0%)	7 (26.9%)	0.010	−0.858
Electrolyte Imbalance	3 (11.5%)	10 (38.5%)	0.025	−0.656
Hypertension	19 (73.1%)	19 (73.1%)	1.00	0.000

**Table 2:** Comparison of admission risk factors, concurrent therapies, and respiratory support modalities.

Variable	VAP-COVID (n = 26)	VAP Non-COVID (n = 26)	*p*-Value	SMD
MDR Risk Factors				
Septic Shock at Admission	4 (15.4%)	16 (61.5%)	<0.001	−1.076
Recent Hospitalization (<3 mo)	3 (11.5%)	8 (30.8%)	0.008	−0.486
Recent Antibiotics (<3 mo)	2 (7.7%)	8 (30.8%)	0.008	−0.613
Concurrent Therapies				
Systemic Corticosteroids	25 (96.2%)	7 (26.9%)	<0.001	2.029
Proton pump inhibitors (PPIs)	22 (84.6%)	5 (19.2%)	<0.001	1.731
Respiratory Support at Admission				
Invasive Mechanical Ventilation	4 (15.4%)	14 (53.8%)	<0.001	−0.882
Non-Invasive Ventilation	18 (69.2%)	1 (3.8%)	<0.001	1.851
Conventional O_2_ Therapy	2 (7.7%)	9 (34.6%)	<0.001	−0.698

**Table 3:** Comparison of radiological patterns, clinical signs at diagnosis, and operative timelines.

Variable	VAP-COVID (n = 26)	VAP Non-COVID (n = 26)	*p*-Value	SMD (95% CI)
Radiological Criteria				
New lung consolidations	5 (19.2%)	26 (100%)	<0.001	−2.901
Progressive consolidations	11 (42.3%)	0 (0%)	<0.001	1.211
New and progressive consolidations	10 (38.5%)	0 (0%)	<0.001	1.119
Clinical Criteria at VAP Diagnosis				
Septic Shock	16 (61.5%)	24 (92.3%)	0.008	−0.785
Worsening Oxygenation	24 (92.3%)	23 (88.5%)	1.00	0.129
Leukocytosis	24 (92.3%)	22 (84.6%)	0.66	0.243
Purulent Secretions	22 (84.6%)	26 (100%)	0.11	−0.603
Clinical Timelines (Mean ± SD)				
Lab Turnaround (hours)	82.35 ± 29.14	67.46 ± 22.55	0.037	0.571 [0.01; 1.14]
Total Hospital Stay (days)	21.08 ± 9.79	47.65 ± 35.60	0.006	−1.018 [−1.63; −0.4]
Total ICU Stay (days)	17.38 ± 7.90	43.31 ± 35.52	0.004	−1.007 [−1.61; −0.39]
Total Mech. Vent. (days)	13.35 ± 5.54	24.24 ± 13.74	0.002	−1.047 [−1.66; −0.42]
VAP Development (days)	6.5 ± 2.78	9.04 ± 8.35	0.562	−0.408 [−0.96; 0.16]

**Table 4:** Microbiological etiology, antimicrobial resistance profiles, and adequacy of empiric therapy. XDR: Extensively Drug-Resistant; MDR: Multidrug-Resistant.

Variable	VAP-COVID (n = 26)	VAP Non-COVID (n = 26)	*p*-Value	SMD
VAP Etiology (n, %)				
*Acinetobacter baumannii*	13 (50%)	10 (38.5%)	>0.05	0.233
*Klebsiella pneumoniae*	8 (30.8%)	9 (34.6%)	>0.05	−0.081
*Pseudomonas aeruginosa*	5 (19.2%)	4 (15.4%)	>0.05	0.101
*Escherichia coli*	0 (0%)	2 (7.7%)	>0.05	−0.408
*Staphylococcus aureus (MRSA/MSSA)*	1 (3.8%)	3 (11.5%)	>0.05	−0.293
Fungal Co-infection (*Candida* spp.)	7 (26.9%)	3 (11.5%)	>0.05	0.399
Resistance Profile (n, %)				
XDR (Extensively Drug-Resistant)	20 (76.9%)	17 (65.4%)	0.35	0.256
MDR (Multidrug-Resistant)	5 (19.2%)	4 (15.4%)	0.71	0.101
Sensitive	2 (7.7%)	5 (19.2%)	0.22	−0.342
Specific Resistance (n, %)				
Carbapenem Resistance	20 (76.9%)	13 (50%)	0.04	0.582
Fluoroquinolone Resistance	23 (88.5%)	16 (61.5%)	0.02	0.656
Piperacillin/Tazobactam Resistance	22 (84.6%)	17 (65.4%)	0.10	0.455
Resistance in Early-Onset VAP	(n = 10)	(n = 11)		
Carbapenem Resistance	8 (80%)	4 (36.4%)	0.043	0.985
Therapy				
Inadequate Empiric Therapy	23 (88.5%)	19 (73%)	0.15	0.401

**Table 5:** Clinical outcomes, mortality timing, and prognostic scores. SOFA: Sequential Organ Failure Assessment; SAPS II: Simplified Acute Physiology Score II; APACHE II: Acute Physiology and Chronic Health Evaluation II.

Variable	VAP-COVID (n = 26)	VAP Non-COVID (n = 26)	*p*-Value	SMD (95% CI)
Overall Mortality (n, %)	21 (80.8%)	23 (88.5%)	0.442	−0.215
Mortality Timing (n, %)				
14–28 days	12 (46.2%)	4 (15.4%)	0.016	0.708
>28 days	2 (7.7%)	14 (53.8%)	<0.001	−1.153
Survival Analysis				
Median Survival (days)	18 (95% CI: 15–27)	45 (95% CI: 27–66)	0.34 (Log-rank)	
Scores at Admission (Mean ± SD)				
SOFA	8.04 ± 3.8	9.2 ± 3.17	0.185	−0.33 [−0.88; 0.23]
SAPS II	44.0 ± 16.54	54.65 ± 13.97	0.011	−0.696 [−1.27; −0.12]
APACHE II	20.66 ± 9.38	22.2 ± 7.52	0.441	−0.181 [−0.73; 0.37]
Charlson Comorbidity Index	4.77 ± 2.16	8.15 ± 2.98	<0.001	−1.298 [−1.94; −0.64]
Dynamic SOFA (Mean ± SD)				
SOFA 2 days before VAP	8.46 ± 2.91	10.85 ± 3.2	0.008	−0.78 [−1.36; −0.19]
SOFA on day of VAP	8.85 ± 3.04	10.12 ± 2.9	0.001	−0.427 [−0.98; 0.14]

**Table 6:** Dynamic Hematological Profile of Deceased Patients at Key Time Points. Data for deceased patients only (n = 21 VAP-COVID, n = 23 VAP non-COVID). NLR: Neutrophil-Lymphocyte Ratio; RDW- SD: Red Cell Distribution Width-Standard Deviation.

Parameter (Median, Q1–Q3)	Group	At ICU Admission	At VAP Diagnosis	At Day 28
Leukocytes (K/µL)				
	VAP-COVID	12.27 (7.68–19.49)	16.43 (12.8–23.02)	12.23 (9.32–22.87)
	VAP non-COVID	13.89 (9.02–17.52)	11.57 (8.96–15.73)	9.86 (7.62–15.25)
	*p*-Value	0.690	0.001	0.148
Neutrophils (K/µL)				
	VAP-COVID	10.82 (6.75–18.48)	15.63 (11.89–22.11)	11.15 (8.66–22.09)
	VAP non-COVID	11.94 (7.51–16.06)	10.04 (7.75–13.51)	7.8 (5.96–11.51)
	*p*-Value	0.953	<0.001	0.042
Lymphocytes (K/µL)				
	VAP-COVID	0.44 (0.26–0.72)	0.46 (0.22–0.68)	0.45 (0.31–0.66)
	VAP non-COVID	0.68 (0.44–1.15)	0.77 (0.48–1.57)	0.97 (0.56–2.34)
	*p*-Value	0.019	0.004	0.002
NLR (Ratio)				
	VAP-COVID	22.6 (11.52–50.29)	41.36 (21.08–74.22)	25.54 (17.5–43.19)
	VAP non-COVID	15.4 (8.57–20.8)	9.63 (5.71–21.89)	5.9 (4.88–11.81)
	*p*-Value	0.068	<0.001	<0.001
Monocytes (K/µL)				
	VAP-COVID	0.38 (0.21–0.61)	0.47 (0.35–0.85)	0.27 (0.1–0.58)
	VAP non-COVID	0.73 (0.38–0.98)	0.49 (0.24–0.73)	0.48 (0.26–0.83)
	*p*-Value	0.003	0.581	0.039
RDW-SD (fL)				
	VAP-COVID	45.4 (42.95–47.15)	47.5 (43.95–50.2)	48.2 (44.9–51.9)
	VAP non-COVID	58.7 (52–63.5)	59.8 (53.7–64.6)	62.6 (52.3–67.5)
	*p*-Value	<0.001	<0.001	<0.001

## Data Availability

The data presented in this study are available on request from the corresponding author. The data are not publicly available due to ethical and privacy restrictions regarding patient health information.

## Abbreviations

APACHE II	Acute Physiology and Chronic Health Evaluation II
CFU	Colony Forming Units
CI	Confidence Interval
COVID-19	Coronavirus Disease 2019
CPIS	Clinical Pulmonary Infection Score
CR-hvKp	Carbapenem-Resistant Hypervirulent *Klebsiella pneumoniae*
CRKP	Carbapenem-Resistant *Klebsiella pneumoniae*
ESCMID	European Society of Clinical Microbiology and Infectious Diseases
GERD	Gastroesophageal Reflux Disease
ICU	Intensive Care Unit
IQR	Interquartile Range
MDR	Multidrug-Resistant
MRSA	Methicillin-Resistant *Staphylococcus aureus*
MSSA	Methicillin-Susceptible *Staphylococcus aureus*
NIV	Non-Invasive Ventilation
NLR	Neutrophil-to-Lymphocyte Ratio
OR	Odds Ratio
PCR	Polymerase Chain Reaction
PDR	Pandrug-Resistant
POC-PCR	Point-of-Care Polymerase Chain Reaction
PPE	Personal Protective Equipment
PPI	Proton Pump Inhibitor
RDW-SD	Red Cell Distribution Width-Standard Deviation
SAPS II	Simplified Acute Physiology Score II
SARS-COV-2	Severe Acute Respiratory Syndrome Coronavirus 2
SD	Standard Deviation
SOFA	Sequential Organ Failure Assessment
VAP	Ventilator-Associated Pneumonia
WHO	World Health Organization
XDR	Extensively Drug-Resistant
